# Management of Mesh Complications after SUI and POP Repair: Review and Analysis of the Current Literature

**DOI:** 10.1155/2015/831285

**Published:** 2015-04-20

**Authors:** D. Barski, D. Y. Deng

**Affiliations:** ^1^Department of Urology, Lukas Hospital, Preussenstrasse 84, 41464 Neuss, Germany; ^2^Department of Urology, University of California, 400 Parnassus Avenue A633, P.O. Box 0738, San Francisco, CA 94143-0738, USA

## Abstract

*Purpose.* To evaluate the surgical treatment concepts for the complications related to the implantation of mesh material for urogynecological indications. *Materials and Methods.* A review of the current literature on PubMed was performed. *Results.* Only retrospective studies were detected. The rate of mesh-related complications is about 15–25% and mesh erosion is up to 10% for POP and SUI repair. Mesh explantation is necessary in about 1-2% of patients due to complications. The initial approach appears to be an early surgical treatment with partial or complete mesh resection. Vaginal and endoscopic access for mesh resection is favored. Prior to recurrent surgeries, a careful examination and planning for the operation strategy are crucial. *Conclusions.* The data on the management of mesh complication is scarce. Revisions should be performed by an experienced surgeon and a proper follow-up with prospective documentation is essential for a good outcome.

## 1. Introduction

Pelvic organ prolapse (POP) affects about 50% of parous women. Approximately, 11% of these women will need surgical correction due to symptoms, like incontinence, voiding dysfunction, and discomfort from vaginal bulge. In the USA, more than 300,000 women undergo surgery for POP annually [1]. Repair with native tissue showed a high recurrence rate up to 30%, especially in the anterior compartment [2]. To reduce the risk of recurrence, transvaginal mesh has been applied in the treatment of POP since the 1990s. In the last decade, the number of mesh operations and various presumed easy-to-use mesh kits from various manufacturers grew exponentially. This development led to a widespread application of this outpatient surgical method. Less attention was paid to possible new complications and only a few clinical trials were available prior to product approval and application. Meshes or grafts potentially add to the complication profile. These include the trauma of insertion, foreign body reaction to the implant in terms of inflammation, infection and/or rejection, contraction of the mesh causing pain, and the stability of the prosthesis over time [3]. In 2008, the U.S. Food and Drug Administration (FDA) issued a warning in dealing with foreign materials for incontinence and POP repair, based on the report of more than 1000 serious side effects by Manufacturer and User Device Experience (MAUDE). Following a systematic review of the literature, the FDA pronounced further examinations on benefits and risks of surgical mesh for SUI (stress urinary incontinence) and POP repair. In September 2011, the FDA organized a scientific advisory board and made 34 manufacturers of POP meshes and 7 manufacturers of SUI meshes perform clinical retrospective studies on their products [4]. Currently, over 30.000 cases due to mesh-related complications and law suits on several manufacturers are brought before the US courts. Reacting to this, several products have been withdrawn from the market by the manufacturers. Despite these developments, in Germany, there are relatively few reactions to the alerts. The changes in the supervision of the medical device approval are currently under debate for the coming EU regulation. In addition to comprehensive education and information of patients on specific mesh-related complications, a special surgical skills training in dealing with foreign materials and the management of possible complications is recommended [5–7].

## 2. Methods

A systematic review was performed for English language articles published in the last five years from January 2009 to June 2014 in PubMed and the Cochrane Library Database. Search items included the following keywords and phrases: “pelvic organ prolapse and POP,” “incontinence,” “vaginal surgery,” “sacrocolpopexy,” “vaginal mesh or implant,” “abdominal mesh or implant,” “alloplastic material,” “Prolift,” “Apogee,” “Perigee,” “Gynemesh,” “Gore-Tex,” “complications,” “vaginal or endoscopic or laparoscopic or abdominal resection,” and “explantation.” Keywords appeared in the title, abstract, or both. Studies with more than 10 reported complications after mesh application for POP or SUI were included. Studies with lacking information on primary surgery, complications, and management were excluded. Classification, risk factors, and treatment concepts of complications after mesh implantation were analyzed. The primary outcomes assessed were the subjective (patient-reported) and objective cure/improvement rates. Secondary outcomes included reoperations for complications and recurrent incontinence after the initial treatment. Data were analysed using RevMan v.5.3 (Cochrane Collaboration, Oxford, UK) and GraphPad Prism v.6 (Graphpad Software, Inc.). Quantitative synthesis was done when more than one eligible study was identified. The outcome results were expressed as weighted means difference (WMD), standard deviations (SDs), and risk ratio (RRs) with 95% confidence intervals (CIs) for dichotomous variables using the Mantel-Haenszel method [8]. Methodological heterogeneity was assessed during selection, and statistical heterogeneity was measured using the chi-square test and *I* ^2^ scores. A random effects model was used throughout to reduce the effect of statistical heterogeneity [9]. Treatment failure risk was defined as reoperation after the initial treatment.

## 3. Results and Discussion

No randomized trials on the surgical treatment of mesh complications were detected. Only one was a partly prospective trial on mesh resection [10]. A total of 17 retrospective studies were included in the review (Table 1). Different conservative approaches and surgical techniques for the resection of alloplastic materials after the treatment of pelvic organ prolapse and stress urinary incontinence are presented. Initial surgeries were midurethral sling (MUS), transvaginal mesh, and abdominal colposacropexy. Only alloplastic polypropylene materials were used.

### 3.1. Classification of Complications

To analyze the mesh-related complications, a Clavien-Dindo classification of surgical operations is often used in the literature [11]. The advantages hereby are a clear correlation to the management of complications and broad acceptance. However, the information on the site and timing of complications is missing. In addition, the classification is not always adequate; for example, the clinically less severe intraoperative bladder injuries must be classified as Grade III complications and distort the analysis. International Continence Society (ICS) and International Urogynecologic Association (IUGA) introduced in 2010 a consensus-based standardized terminology and classification for the description and documentation of specific complications after the use of implants in pelvic floor surgery of women [3]. The classification is based on the information on the category, time, and location of complications. Because of high complexity and low concordance in different trials, the ICS/IUGA classification is currently rarely used [6, 12]. However, the classification could be valuable for the reporting of long-term data in registries.

### 3.2. Complications and Risk Factors

Polypropylene meshes are usually used for vaginal repair of POP and SUI. The overall rate of mesh-related complications after transvaginal mesh application for POP is about 15–25% and mesh erosion is up to 10% for these indications [6, 13]. The most common complications (retrospective review of 388 cases with complications) after implantation of midurethral sling (MUS) are overactive bladder (52%), obstructive micturition (45%), SUI (26%), vaginal mesh exposure (18%), chronic pelvic pain (14%), local infection (12%), dyspareunia (6%), and vesicovaginal fistula (4%) ([14], Table 2). Kasyan et al. analyzed the biggest series of 152 complications (22.5%) following Prolift transvaginal mesh for POP. The following complications were detected: erosions (21%), dyspareunia (11%), mesh shrinkage (4.4%), pelvic abscess (2.7%), and fistula (1.3%). Younger age, less prominent prolapse, hematomas, and concomitant hysterectomies were associated with higher risk of complications [15]. As part of the abdominal sacrocolpopexy where nonabsorbable synthetic materials (Mersilene, Prolene, Polypropylene, Gore-Tex) are applied, the risk for mesh erosion is between 0 and 12% (medium risk 4%). Causes of complications were primarily surgical techniques, concomitant surgeries, non-type 1 meshes, and previous surgery in the field [6, 7, 16]. Most complications occur in a time range of one to five years after the operation [12]. Median time to revision in selected trials was 19.2 mos (5.8–59). The complications are attributed to a considerable extent to the wrong indication, faulty surgical techniques (tape positioning and overcorrection), and material properties (biocompatibility and contraction of the mesh material). New developments in material optimization are currently expected. Other risk factors retrieved from multivariate analysis were previous anti-incontinence procedure, obesity, and estrogen status [5, 6, 15]. Reasons for vaginal mesh exposure of the mesh material are categorized into tissue causes and biomechanical mesh properties. Tissue causes include superficial placement, traumatic dissection, tissue healing, and thin and atrophic vaginal mucosa, especially in postmenopausal women [16].

### 3.3. Management Strategies for Mesh Complications

**Figure 1:**
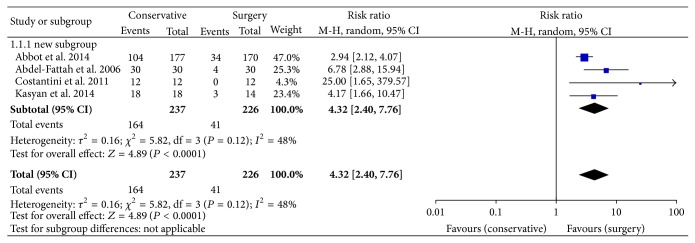
Treatment failure risk for mesh-related complication after conservative treatment versus mesh excision. CI: confidence interval; M-H: Mantel-Haenszel [15–17, 20].

**Figure 2:**
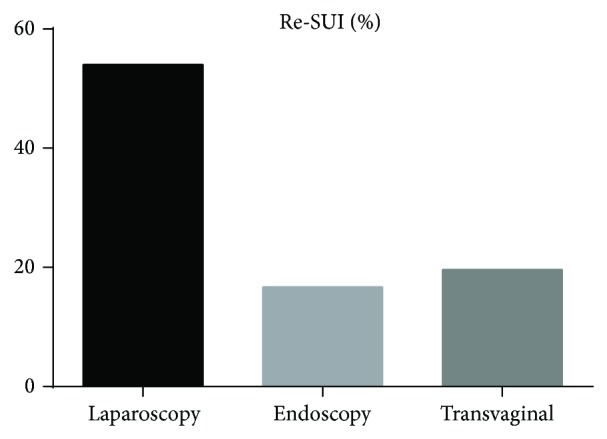
Recurrent incontinence after MUS-mesh excision (mean), *P* < 0.05.

The current retrospective data on mesh excision for complications is presented in Table 1. 12 trials reported on complications after MUS, 8 trials on complications after transvaginal mesh for POP repair, and 3 trials on abdominal colposacropexy. Median patient number in the studies was 42 patients (8–347). Mean follow-up after the treatment of mesh-related complications was 22.6 mos (6 weeks–65 mos). Many authors propagate an initial conservative approach with antibiotics and local estrogen application in cases of mesh erosion. However, new studies show an advantage of the timely revision surgery to relieve the symptoms. The analysis of trials comparing conservative treatment with surgery for mesh erosions showed a 4.32-fold risk ratio for treatment failure after the conservative approach (Figure 1). Abbott and colleagues showed that 60% of the initially conservatively treated patients required surgical intervention and 60% of the total cohort were operated on at least twice [17]. Erosions in the vagina or internal organs with consecutive infection, pain, dys- or hispareunia, voiding dysfunction due to obstruction, and urge incontinence often require surgical revision [25]. In the current US-American and European studies with long-term observation, the rate of postoperative mesh explantations was about 1% after a midurethral sling (MUS) and about 3% after a vaginal mesh for POP repair [26, 32]. The complications can be often corrected by mesh resection, but, in some cases, further surgeries for de novo incontinence (10–25%) or POP (7–47%) were necessary [17].  Figure 2 shows the percentage of recurrent stress incontinence depending on different MUS-excision techniques. Laparoscopic abdominal resection causes a 3-fold higher risk of Re-SUI probably due to a complete incision and excision of the mesh arms [30]. The result was however not significant due to a small trial number. There are a few data on the effect of mesh explantation on dyspareunia and chronic pelvic pain. Previous studies suggest that the pain due to the scarring and foreign body reaction may persist even after the mesh removal [33].

**Figure 3:**
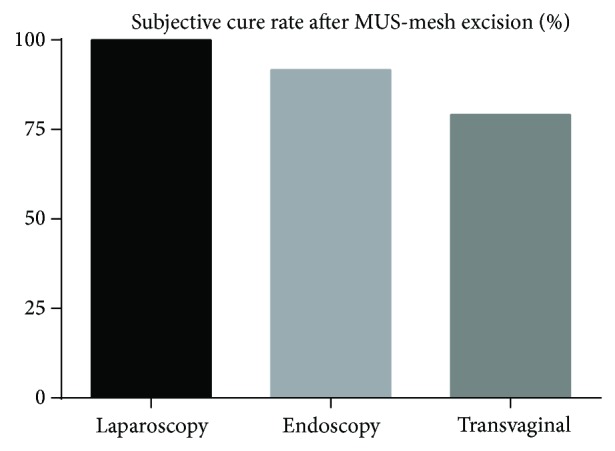
Subjective cure rate after MUS-mesh excision (mean), *P* < 0.05.

A comprehensive diagnosis of symptoms and localization of erosion by cystoscopy, vaginal examination, imaging and urodynamics, education of patients on possible irreversible damage, and careful planning of the operation steps are required prior to revision surgery. A careful clinical examination and determination of the pain location by trigger points are excellent markers for planning of the site and extent of mesh resection [20, 33]. However, a standardized surgical procedure and access do not exist up to date. The analysis of the available studies showed a similar subjective cure rate of 79–100% for different techniques (Figure 3). The rate of reoperations was higher if an endoscopic or transvaginal access were chosen [18, 19, 21, 22, 24, 30]. However, the hospital stay, operation time, and postoperative pain were higher in the case of laparoscopic mesh excision [30]. Generally, a vaginal access with partial or complete resection of the infected foreign material is favored in most trials (88% of the analysed studies). Non-type 1 alloplastic materials according to Amid classification (e.g., polytetrafluoroethylene and Gore-Tex) have to be removed completely in case of erosion or infection in order to achieve symptom relief [34]. A complete mesh excision can be very difficult especially for abdominal access. Complications such as bleeding, fistula, neuropathies, and prolapse recurrence are frequent [20]. Different transvaginal techniques like sling loosening, mesh incision, and partial or complete excision were described in included studies but no clear strategy or algorithm could be found (Table 1). Costantini and colleagues propose the following intraoperative management of mesh exposure: closure of the vaginal defect with double-layer suture to avoid a direct mesh contact with the mucous membranes, flush with antibiotic solution, no stitching of the full thickness of the vaginal wall, atraumatic preparation, use of nonwoven, nonabsorbable suture and polypropylene meshes, avoidance of concomitant hysterectomy, and long-term follow-up after the revision [20]. Similar vaginal techniques with optional excision of the alloplastic material and two-layer closure of a vesicovaginal fistula are described by other authors [22]. The German group from Mainz University reported on the urogynecological management of complications based on 259 patients after implantation of MUS [25]. In the case of de novo OAB, the symptoms improved only after the resection of the portion of the sling which was in contact with the urethra. The wrong position of the sling could be detected by pelvic floor sonography (PFS). PFS is an important tool to assess the tape position, form, and distance from urethra. The reasons for the complications and sling failure can be identified and corrected. The ultrasonography evaluation of a well-positioned sling provides certainty that a success of conservative therapy can be expected. In case of a dystopic position of the sling, the first step is to evaluate the sling location and to decide whether or not the band can be saved [34]. The removal of the foreign material was more difficult if the initial operation has been long ago. Particularly difficult and traumatic for the pelvic floor were the excisions of transobturator tapes [25]. Infections of the alloplastic material in the obturator fossa are especially dangerous for the development of abscesses or necrotising fasciitis and require careful debridement and follow-up. If a significant erosion of the mesh was diagnosed, partial vaginal material removal has been usually performed. In case of vaginal mesh exposure (small erosions under 1 cm without infection), the defect could be closed by a suture. In case of mesh shrinkage, a resection of the fibrotic band in the paravaginal sulci was proposed. In some cases, infection of TOT required extensive debridement with opening of the deep tissues of the groin and adductor compartment, removal of the complete tape, antibiotics, and sometimes hyperbaric oxygen therapy [15]. Agnew and colleagues reviewed 63 women with voiding dysfunction (>150 mL residual volume) after MUS (67% TVT). Three different surgical procedures were analysed (simple sling division, partial resection, and concomitant SUI procedure). Taking into account the results of the findings (Table 1), the authors changed their strategy to divide synthetic midurethral slings lateral to the urethra and then carefully perform cystourethroscopy to ensure that no urinary tract injury has occurred [18].

A tertiary center in the US presented retrospective data on 47 women after salvage operation following at least one revision on mesh-related complications. Different operative strategies and approaches were applied, depending on the intraoperative findings. The median follow-up was 2 years. Patients presented with various symptoms and 72% could be treated successfully (QoL questionnaire) by the first salvage operation. However, 14 women needed a reconstruction of the urethra, 5 women a continent stoma, and 2 women a partial cystectomy. The treatment of patients with symptoms of chronic pain was difficult; only 28% reported a relief of symptoms postoperatively. The authors assume 3 potential causes of mesh-related urethral complications; namely, (1) the surgeon simply pulls the sling too tight at surgery, (2) a correctly placed sling contracts with time due to tissue ingrowth, and (3) faulty surgical technique results in placement of the sling directly into the urinary tract [19].

Other case reports showed good postoperative results after covering the exposed alloplastic material with vulvar fat without resection [35]. In case of sling erosion into the bladder with consecutive infections, stone formation, and pain, transurethral resection or laser excision (holmium and thulium) techniques have been successful [21, 36]. Other groups reported successful individual cases with laparoscopic and robot-assisted excision and transvesical reconstructions to treat the mesh erosions after MUS implantation [30, 37, 38].

## 4. Conclusion

Mesh-related complications are a current emerging problem, which confronts all urologists and gynecologists in their daily practice. The previous findings from retrospective studies show that early surgical treatment of these complications is advantageous. There is no profound evidence based algorithm on the access and surgical procedure up to date. However, transurethral and vaginal mesh excision techniques were demonstrated to be safe and successful in present studies. It is important to ensure a gentle tissue dissection and continuous follow-up after the surgery. The revision operations belong in the hands of experts and should be documented prospectively in trials and registries.

## Figures and Tables

**Table 1:** Studies on management of mesh related complications after incontinence and prolapse surgeries.

Author	Trial	Number of patients	Mesh	Complications	Median time to revision	Management	Concomitant procedure	Follow-up
Abbot et al. 2014 [17]	RT	347 (49.9% MUS; 25.6% TVM or CSP; 24.2% combination)	Various	30% dyspareunia 42.7% mesh erosion 34.6% pelvic pain 77% grade 3 or 4 (reoperation) complication	5.8 mos (0–65.2 mos)	(1) Trimming of mesh/partial excision (50.9%) (2) Release of mesh arms (18.1%) (3) Complete intravaginal mesh excision (26.9%) (4) Recurrent prolapse treatment (23.2%) (5) Recurrent incontinence treatment (14.8%) (6) Other surgeries (20.1%) (7) Initial conservative treatment (23%) 60% ≥2 interventions	MUS	

Agnew et al. 2012 [18]	RT	63 MUS	Various synthetics (67% monofilament TVT, 17% TOT)	100% voiding dysfunction	12.4 mos (1 week–8 yrs)	(1) Simple sling division (73%) (2) Partial excision of sling (21%) (3) Concomitant procedure to prevent Re-SUI (4/63)	Burch, MUS	Persistent voiding dysfunction (1) 10.9%; (2) 7.7%; (3) 50% (*P* = 0.09) Subsequent surgery for recurrent SUI (1) 2.2%; (2) 23.1%; (3) 0% (*P* = 0.04) De novo urgency (1) 10.9%; (2) 15.4%; (3) 25% (*P* = 0.51)

Blaivas et al. 2013 [19]	RT	47 MUS	Type 1 76% Types 2–3 23%	OAB (70%) SUI (55%) Recurrent UTI (21%) Pelvic pain/dysuria (34%) Obstructive symptoms (9%) Vaginal extrusion (9%)	2 yrs (1 mos–8 yrs)	(1) Sling excision + urethrolysis (34%) (2) Sling excision + urethral reconstruction (including fistula repair) + autologous fascial sling (30%) (3) Sling incision (21%) (4) Partial cystectomy (10%) (5) Ureteroneocystostomy (4%)	MUS	2 yrs (0.25–12 yrs) Successful treatment 72% 28% recurrent surgery refractory pain (19%), mesh extrusion (17%), and OAB (8%)

Costantini et al. 2011 [20]	RT	12 (12/179, 6.7%) mesh erosion after abdominal CSP	11 PP, 1 Gore-Tex	100% mesh erosion 41% vaginal bleeding 33% asymptomatic 17% dyspareunia 17% infection (1x Gore-Tex)	22.9 mos (2–66 mos)	(1) Antibiotics and local estrogen (100%) (2) Vaginal (partial) mesh resection (83%) (3) Abdominal resection (17%) (4) Endoscopic (8%)		57 mos (18–120 mos) (1) All needed surgery (3) Recurrent cystocele (4) Fistula, abdominal revision

Davis et al. 2012 [21]	RT	12 TVT	PP	100% mesh erosion	59 mos (7–144 mos)	Endoscopic holmium: YAG laser excision (100%)		65.5 mos (6–134 mos) 33% second laser excision 17% surgery for recurrent SUI 8% (1 patient) abdominal mesh resection

Firoozi et al. 2012 [22]	RT	23 TVM for POP	Various PP	Vaginal/pelvic pain (39%), dyspareunia (39%), vaginal mesh extrusion/exposure (26%), urinary incontinence (35%), recurrent pelvic organ prolapse (22%), bladder mesh perforation (22%), rectal mesh perforation (4%), ureteral perforation injury (4%), and vesicovaginal fistula (9%)	10 mos (1–27 mos)	(1) Transvaginal excision (90%) (2) Transvaginal/endoscopic (5%) (3) Transrectal/transperineal (5%) (4) Concomitant POP/SUI repair (45%)	TVM, MUS	3 mos 14% UTI 4.3% collagen injection for Re-SUI 4.3% PFT for perineal pain

Greiman and Kielb 2012 [23]	RT	28 (28/118, 23%) MUS	PP	Intravesical sling (4%), extruded vaginal mesh (93%), obstructive voiding symptoms (78%), dyspareunia (42%), and vaginal bleeding (21%)	15 mos	(1) Sling loosening, incision in the midline (2) If mesh erosion >1 cm a resection		11% reoperation for mesh extrusion, no other complications

Hammett et al. 2014 [24]	RT	57 patients (26 MUS, 23 TVM, and 9 intraperitoneal prolapse CSP)	Various PP	100% mesh erosion with pelvic pain (55.9%), dyspareunia (54.4%), and vaginal discharge (30.9%).		(1) Vaginal mesh excision (91%) (2) Abdominal resection (all CSP, *n* = 9/15, 40%)		6 weeks 57% symptoms completely resolved 12% required more than 1 surgery for mesh excision (1) 9% UTI (2) 4.5% cardiopulmonal complications; 18% sepsis; 45% wound infection

Hampel et al. 2009 [25]	RT	48 MUS (44 TVT, 4 TOT)	Various PP	De novo urge (65%), mesh erosion (21%), dyspareunia (19%), UTI (35%), and fistula (6%)		(1) Partial mesh resection (trans-/suburethral, 23%) (2) Self-catheterisation (23%) (3) Botox/neuromodulation (27%) (4) Fascia plastic (10%) (5) Complete abdominal-vaginal mesh resection (8%) (6) Urinary diversion (2%) (7) Fistula repair (6%) (8) Conservative treatment (25%)		42% symptoms completely resolved

Kasyan et al. 2014 [15]	RT	152 TVM	Prolift (Gynecare), PP	Erosions (21%), dyspareunia (11%), mesh shrinkage (4.4%), pelvic abscess (2.7%), and fistula (1.3%)		(1) Conservative treatment with local oestrogen (2) Partial/total mesh excision		

Nguyen et al. 2012 [26]	RT	82 MUS (2.2%)	Various			(1) Sling loosening or transaction for voiding dysfunction (60%) (2) Excision for vaginal mesh exposure 30 (36%) (3) Excision for pain (1.2%) (4) Excision for urethral erosion (1.2%) (5) Drainage of retropubic hematoma (1.2%)	MUS, colporrhaphy, and CSP	

Abdel-Fattah et al. 2006 [16]	RT	34 TVM (2.2%)	Various			(1) Excision for vaginal mesh exposure (85%) (2) Excision of vaginal suture (6%) (3) Biologic graft reoperation (12%) (4) Drainage hematoma/abscess (6%) (5) Bowel resection for obstruction (3%)		

Padmanabhan et al. 2012 [27]	RT	85 (MUS, TVM)	Various PP	Perforation of urethra (14%), bladder (36%), and vagina (50%)		(1) Vaginal excision (14%) (2) Lower urinary tract excision (47%) (3) Partial cystectomy (21%) (4) Urethroplasty (21%)		Subjective cure in 75% and improvement in 21% SUI (6.6–12.5%)

Renezeder et al. 2011 [28]	RT	118 (80% MUS, 20% TVM)	Various PP (88% type 1)	De novo urgency (46.6%), dyspareunia (41.5%), recurrent UTI (39.0%), mesh erosion (37%), and vaginal bleeding (9.3%)	27 mos (1–89 mos)	(1) Tissue patch covering (17.8%) (2) Partial removal (65.3%) (3) Complete removal per laparotomy (12.7%) (4) Bone stabilization (0.8%) (5) Excision of granulation tissue (3.4%)		8 weeks 45.5% urgency

Ridgeway et al. 2008 [29]	RT	19 TVM	Monofilament PP	Chronic pain (31%), dyspareunia (31%), recurrent pelvic organ prolapse (42%), mesh erosion (63%), and vesicovaginal fistula (16%)		Partial tailored vaginal mesh resection with concomitant procedures	Burch, MUS	33 weeks (16–75 weeks) 16% UTI 5% hematoma 21% persistent symptoms

Rouprêt et al. 2010 [30]	RT	38 TVT	PP	Mesh erosion/extrusion (42%), pelvic pain (39%), and obstruction (18%)		(1) Laparoscopic (97%) (2) Laparoscopic + vaginal (3%)		38 mos (2–80) Healing and pain release (100%) Recurrent SUI (66%)

Shah et al. 2013 [31]	RT	21 MUS	Polypropylene, type I	Urethral perforation (67%), bladder perforation (33%), fistula (19%), vaginal pain (67%), urgency (29%), incontinence (38%), obstruction (33%), dyspareunia (19%), and hematuria (24%)	15.5 mos (1–60 mos)	(near) Total mesh excision, urinary tract reconstruction, and concomitant pubovaginal sling with autologous rectus fascia	MUS, urethroplasty	22 mos (6–98 mos) Continence (81%) Incisional seroma (9.5%) Additional procedures (36%) UTI (9.5%) Pelvic pain (9.5%) dyspareunia 9.5%

RT: retrospective trial; PT: prospective trial; MUS: midurethral sling; TVM: transvaginal mesh; TVT: tension-free vaginal tape; TOT: transobturator tape; CSP: colposacropexy; PP: polypropylene.

**Table 2:** Complications of midurethral slings (total number: 388 women sent for revision) [14].

Complications	Number	Percentage
Overactive bladder	201	51.8%
Lower urinary tract obstruction	173	44.58%
Recurrence of SUI	101	26.03%
Vaginal exposure	68	17.52%
Pain	54	13.91%
Infective complications	48	12.37%
Dyspareunia	22	5.67%
Vesicovaginal fistula	14	3.6%
Inrolled sling or contraction of material	18	4.63%
Intraoperative bladder injury	11	2.83%
Groin/upper thigh pain	11	2.83%
Postoperative hematoma	10	2.57%
Bladder/urethral penetration	18	4.63%
Foreign body sensation in vagina	6	1.54%
Husband's penis laceration	6	1.54%
Groin infection	4	1.03%
Necrotizing fasciitis	3	0.77%
Retropubic abscess	3	0.77%
Urethrovaginal fistula	2	0.51%
Intraoperative bowel injury	1	0.25%
